# The Effect of Dental Adhesive Composition and Etching Mode on Microleakage of Bonding Agents in Primary Molar Teeth

**DOI:** 10.30476/DENTJODS.2021.90489.1497

**Published:** 2022-09

**Authors:** Baharan Ranjbar Omidi, Soolmaz Heidari, Fatemeh Farahbakhshpour, Elham Tavakolian Ardakani, Monirsadat Mirzadeh

**Affiliations:** 1 Dept. of Operative Dentistry, Dental Caries Prevention Research Center, Qazvin University of Medical Sciences, Qazvin, Iran; 2 Dept. of Pediatric Dentistry, Faculty of Dentistry, Qazvin University of Medical Sciences, Qazvin, Iran; 3 Dept. of Community Medicine, Metabolic Disease Research Center, Research Institute for Prevention of Non-Communicable Diseases, Qazvin University of Medical Sciences, Qazvin, Iran

**Keywords:** Dental leakage, Dental etching, Tooth, Deciduous, Molar

## Abstract

**Statement of the Problem:**

The dilemma of microleakage at the composite-tooth interface is still a major challenge in operative dental practice.

**Purpose:**

This study aimed to compare the microleakage of universal adhesive with self-etch and total-etch bonding strategies for restoration of class-II primary molar cavities.

**Materials and Method:**

This in vitro, experimental study was conducted on 75 extracted primary molars. Class-II cavities were prepared in mesial or distal surfaces. The teeth were randomly divided into five groups of Adper Single Bond 2 (3M ESPE; St Paul, MN, USA), Clearfil SE Bond (Kuraray Noritake, Osaka, Japan), G-Bond (GC Corp., Tokyo, Japan), G-Premio Bond (GC Corp., Tokyo, Japan) with total-etch mode and G-Premio Bond with self-etch mode. Cavities were also restored with Nano-hybrid resin composite (Grandio, VOCO, Cuxhaven, Germany) and incubated for 24 hours, followed by thermocycling at 1500× between 5-55°C within a dwell time of 20 seconds. Later, the cavities were placed in 1M silver nitrate solution and evaluated under a stereomicroscope. Finally, microleakage was assessed quantitatively and qualitatively. One tooth in each group was prepared and evaluated under a scanning electron microscope (SEM). Data were analyzed using One-way ANOVA, Tukey's post hoc test and Chi-square test (*p*< 0.05).

**Results:**

The microleakage values were significantly different in the study groups (*p*< 0.05). The highest level of microleakage was noted in G-Bond and the lowest in G-Premio Bond with total etching. There was a significant correlation between the qualitative and quantitative measurements of microleakage.

**Conclusion:**

The G-Premio Bond yielded acceptable results in terms of microleakage in total-etch and self-etch modes. However, additional etching is recommended to improve the quality of bonding.

## Introduction

In pediatric dentistry, restoration of tooth decay with tooth-colored restorations is one of the most common treatments [ 1 ]. Dental adhesive agents provide a seal between composite restoration and tooth structure. Despite recent advances in this regard, the microleakage at the composite-tooth interface remains a major problem that results in marginal discoloration, secondary caries, and subsequent loss of retention [ 1 ]. Thus, one of the main factors affecting the clinical success of restorations is the proper bonding of composite to dentin and enamel [ 1 - 2 ]. 

In recent decades, two different bonding strategies namely etch and rinse approach and self-etch systems were introduced for dental bonding purpose [ 2 ]. Etch and rinse adhesives are used in two or three steps. After the removal of smear layer and demineralization of dentin and enamel, dentin collagen fibrils are exposed to enable and facilitate adhesive penetration into porosities [ 2 ]. The self-etch adhesive systems are used in one or two steps. The main component of self-etch systems is composed of aqueous solution of functional monomers with a higher pH compared to phosphoric acid etchants [ 3 ].

One major problem in self-etch bonding system is the weak bonding to enamel margins, which is highly dependent on the adhesive pH [ 4 ]. This problem was later suggested to be solved by additional etching of enamel; however, controversies still exist on the efficacy of additional etching of enamel prior to the application of self-etch primer [ 4 ]. Manufacturers recently introduced universal adhesives, also known as multimode or multipurpose adhesives, which can be used with both etch and rinse, and self-etch strategies. One of the properties of universal dental adhesives is the ability to be used with any etching procedure. This is due to the presence of carboxylate and phosphate monomers in their composition that makes the chemical bonding to hydroxyapatite possible [ 3 ].

It has been shown that enamel pre-etching significantly improves the bond strength of universal dental adhesives [ 5 ]. On the other hand, Suzuki *et al.* [ 6 ] showed that self-etch mode provides sufficient enamel bond.

Microleakage assessment by dye penetration is one of the most commonly used methods due to its simplicity and quickness [ 7 ]. Conventionally, an expert performs the visual assessment using a microscope; however, it is highly subjective and it might decrease the accuracy of assessment [ 7 ]. To increase accuracy, quantitative methods are used to evaluate the morphometry of specimens. In this method, microleakage is assessed using an image processing software and the results are reported in microns [ 7 ]. However, this method is time-consuming. Accordingly, this study has used both techniques to measure the amount of microleakage.

Concerning the insufficient information about the performance of these adhesives in primary teeth, this study aimed to assess the microleakage of class-II composite restorations of primary molars using etch and rinse, self-etch, and multi-mode universal adhesive. 

## Materials and Method

This study was approved by Ethical Committee of Qazvin University of Medical Sciences with ethical number of IR.QUMS.REC.1395.70. 

This in vitro experimental study was conducted on 75 extracted primary molars. The sample size was calculated to be 15 teeth in each group with an alpha error = 0.05, power = 80% and significance level = 0.05. 

Extracted primary molars with a minimum of one sound proximal surface were chosen. After debridement, the teeth were immersed in 0.5% chloramine T solution for seven days followed by immersing in distilled water at room temperature. 

Standard class-II cavities with 3mm buccolingual dimension, 1.5mm axial wall depth, and 3mm cavity height were prepared in the mesial or distal surfaces (determined randomly). The gingival wall was located in enamel. Based on the bonding system used, five groups were marked as follows. Table 1 shows the composition of bonding agents used in this study. 

**Table 1:** Composition of bonding agents used in this study

Material	Composition	Manufacturer
Adper single bond 2	Etchant: 35% H_3_PO_4_; pH: 0.7 Adhesive: Bis-GMA, dimethacrylates, HEMA, poly alkenoic acid copolymer, 5 nm silica fillers, ethanol, water, photoinitiator	3M ESPE, St Paul, MN, USA
Clearfil SE bond	Primer: MDP, HEMA, photoinitiator, Water, hydrophilic dimethacrylate; Adhesive: MDP, HEMA, Bis-GMA, hydrophobic dimethacrylate, submicron silica fillers, photoinitiator, n,n,diethanol-p-toluidine; pH: 2	Kuraray Noritake Dental, Tokyo, Japan
G-bond	4-MET, phosphoric acid ester-monomer, UDMA, TEGDMA, acetone, water, stabilizer, silica fillers, photoinitiator; pH: 2.8	GC Corp, Tokyo, Japan
G-Premio bond	MDP, MDTP, 4-MET, BHT, acetone, water, dimethacrylate monomer, photoinitiator, silica fillers; pH: 1.5	GC Corp, Tokyo, Japan

Bis-GMA: bisphenol A-glycidyl methacrylate, HEMA: hydroxyethyl methacrylate, MDP: methacryloyloxydecyl dihydrogen phosphate, MDTP: methacryloyloxydecyl dihydrogen thiophosphate, 4-MET: 4-methacryloyloxy ethyl trimellitic acid, UDMA: urethane dimethacrylate, TEGDMA: triethylene glycol dimethacrylate, BHT: butylated hydroxytoluene

• Group 1: Adper Single Bond 2 as control group (etch and rinse)

• Group 2: Clearfil SE Bond (self-etch, two components) 

• Group 3: G-Bond (self-etch, one component)

• Group 4: G-Permio Bond (multi-mode universal bonding agent with total etch mode)

• Group 5: G-Premio Bond (multi-mode universal bonding agent with self-etch mode)

All bonding agents were applied according to the manufacturers’ instructions. 

In Adper Single Bond 2 group, after cavity preparation, etching with 38% phosphoric acid (Etch-Rite, PulpDent, USA) was applied for 20 seconds. Then, the cavity was rinsed, gently dried, and received two layers of bonding agent. The bonding agent was air-dried for 5 seconds, and light cured (Woodpecker, Guiliin, China) at a light intensity of 800mW/cm2 for 10 seconds.

In Clearfil SE Bond group, after cavity preparation, the primer was applied on the surface for 20 seconds, dried with mild air flow, and then, one coat bonding agent was applied and finally, after a gentle air flow, it was cured for 10 seconds.

In G-Bond group, after cavity preparation and application of bonding agent to gently dried cavity, it was left undisturbed for 5-10 seconds and then, the cavity was dried at the maximum air pressure followed by 10 seconds of curing.

In G-Premio Bond group without any separate etching, the bonding agent was applied on the gently dried cavity surfaces and left for 10 seconds, dried at maximum air pressure for 5 seconds and cured for 10 seconds. In the G-Premio Bond group with separate etching, 10-15 seconds of etching was performed, followed by rinsing prior to the application of bonding agent. After the application of bonding agent to all surfaces, the cavities were restored with a universal nano-hybrid composite (Grandio, Voco, Cuxhaven, Germany) with 2mm thick increments. Each layer was light cured for 20 seconds. After completion of cavity restoration, it was polished with composite finishing and polishing burs. The apices of teeth were sealed with composite, immersed in distilled water, and kept at 37°C for 24 hours. The samples were then subjected to 1500 thermal cycles between 5-55°C with a dwell time of 20 seconds and transfer time of 20 seconds. Later, all specimens were covered with two layers of nail varnish up to 1mm around the restoration margin. After that, the teeth were immersed in 1M silver nitrate (17g in 100cc of distilled water) solution for 6 hours, then, in the developer solution for 12 hours, followed by exposure to fluorescent light for 6 hours. The teeth were mounted in clear acrylic resin and sectioned longitudinally in a mesiodistal direction using low speed diamond disk (Mecatome T201A, Presi, France) under water coolant. The sectioned specimens were evaluated for microleakage under a stereomicroscope (LEICA, EZ4D, Singapore) at 10× magnification. Assessment of microleakage at the tooth-restoration interface was done using the following qualitative and quantitative classifications:

### Qualitative assessment

Microleakage was scored as (0) no dye penetration, (1) dye penetration extending to the enamel and maximally to one-third of gingival dentinal floor, (2) dye penetration extending to more than one-third of gingival dentinal wall, but less than half of it, (3) dye penetration extending to the entire gingival dentin floor, but not reaching the axial wall and dental pulp, and (4) dye penetration extending to the entire gingival dentin floor and axial wall and dental pulp [ 8 ].

### Quantitative assessment

**Figure 1:**
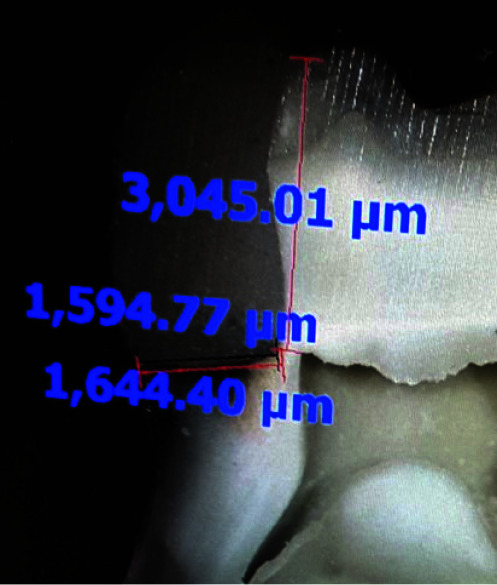
Quantitative assessment of microleakage in the gingival and axial walls

Microleakage levels of sectioned teeth were checked by 10× magnification under a stereomicroscope. The amount of microleakage was measured in microns in gingival and axial walls. To calculate the total microleakage (MT%), the sum of gingival and axial microleakage (MG and MA) levels was divided by the sum of two wall lengths (LG and LA) (Figure 1) [ 2 ].

$\mathrm{MT\%}=\frac{\mathrm{MG}+\mathrm{MA}}{\mathrm{LG}+\mathrm{LA}}\times 100\%$

### Scanning electron microscope (SEM)

To evaluate the morphology of resin-enamel interface, one sample was selected from the control group and also from the groups with the highest and lowest microleakage. Samples were first immersed in 6N hydrochloric acid for 30s. After rinsing with water for 5 minutes, the teeth were immersed in 2.5% sodium hypochlorite solution for 5 minutes and rinsed again under running water for 5 minutes [ 9 ]. 

The specimens were then dried and sputter coated with gold and their morphology was determined under a SEM (XL30 ESEM, Philips, Poland) with an accelerated voltage of 24Kv.

### Statistical analysis

All data were analyzed using a statistical software package (SPSS version 21). One-way ANOVA and Tukey's post hoc test were applied to analyze quantitative data. Chi-square test was used to compare microleakage scores between studied groups. In all tests, *p*< 0.05 was considered statistically significant.

## Results

The quantitative data of this research demonstrated that the highest degree of microleakage was in G-Bond group and the lowest degree was in G-premio bond with etching group. Table 2 shows the mean and standard deviation of microleakage in the five study groups. The mean microleakage in Clearfil SE Bond and G Bond was significantly higher than that of G-Premio Bond with etching. Furthermore, the mean microleakage of G-Premio Bond without etching was significantly higher than that of G-Premio Bond with etching. Finally, the mean microleakage in G Bond was significantly higher than that of Adper Single Bond 2. No other significant differences were noted (Table 3).

**Table 2:** Mean microleakage in the five groups (MT%)

Bonding agent	Mean	SD	*p* Value
Clearfil SE Bond	13.5	11.7	0.001
G-Premio Bond with etching	4.9	9.7	
Adper Single Bond 2	8.5	10.2	
G-Bond	17.7	14.8	
G-Premio Bond without etching	13.8	13.5	

**Table 3:** Pairwise comparison of the groups in terms of microleakage

Group		*p* Value
Clearfil SE Bond	G-Premio Bond with etching	0.002
Clearfil SE Bond	Adper Single Bond 2	0.096
Clearfil SE Bond	G-Bond	0.25
Clearfil SE Bond	G-Premio Bond without etching	0.88
G-Premio Bond with etching	Adper Single Bond 2	0.06
G-Premio Bond with etching	G-Bond	0.00
G-Premio Bond with etching	G-Premio Bond without etching	0.003
Adper Single Bond 2	G-Bond	0.012
Adper Single Bond 2	G-Premio Bond without etching	0.13
G-Bond	G-Premio Bond without etching	0.33

**Figure 2:**
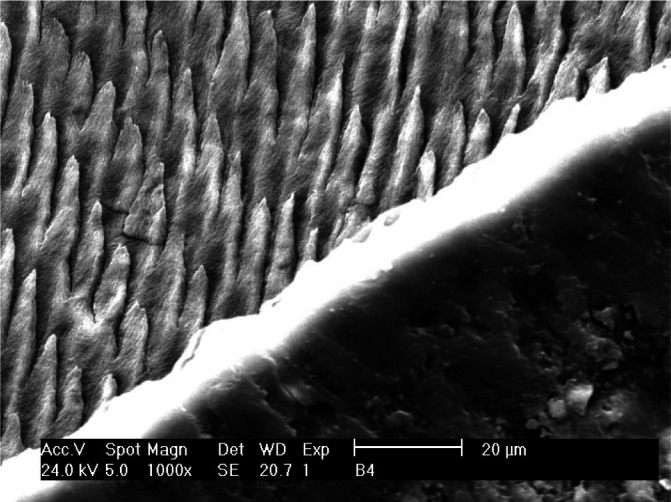
Enamel-composite interface after the application of G-Premio Bond with etching

**Figure 3:**
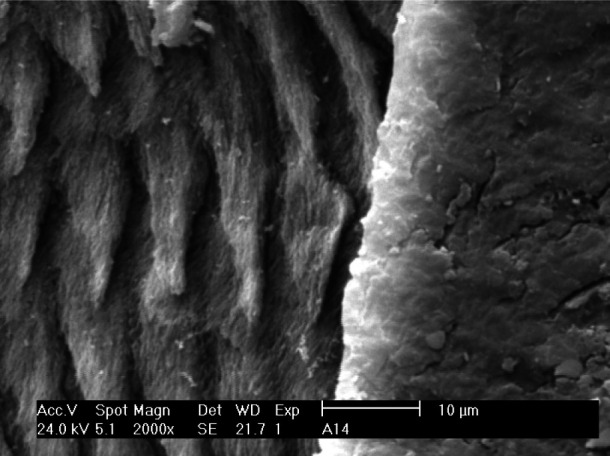
Enamel-composite interface after the application of Adper Single Bond 2

**Figure 4:**
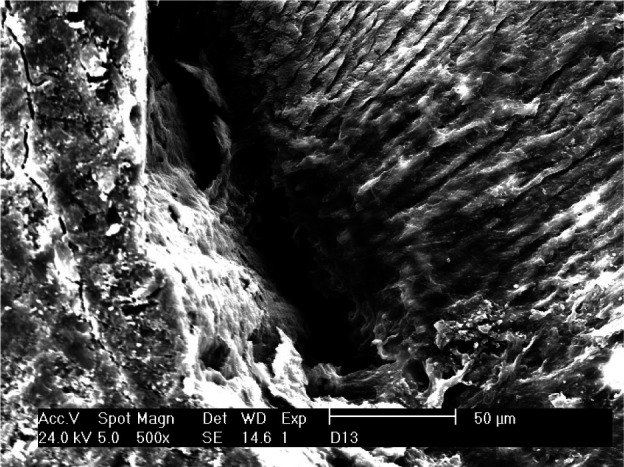
Enamel-composite interface after the application of G Bond

Table 4 shows the relative frequency of microleakage of Grandio composite by the application of different boding agents based on qualitative analysis. In our study, in qualitative assessment, the highest score of microleakage (score 3) was noted in G-Bond (6.7%), while this group showed the lowest number of teeth without microleakage (23.3%). G-Premio universal adhesive with etching revealed the highest percentage of teeth without microleakage (73.3%), which was in agreement with quantitative results (*p*= 0.004). There was also a strong significant positive correlation between the microleakage qualitative and quantitative variable scores using Spearman's rank correlation coefficient (*p*< 0.001, r= 0.96). In SEM images, etch patterns of Adper Single Bond 2 and G-Premio Bond with etching were similar (Figures 2 and Figure 3). Etch pattern in G-Bond was mild and enamel rods were not exposed as clear as two previous adhesives. A distinct gap at interface region was noted in G-Bond (Figure 4).

**Table 4:** Relative frequency of microleakage of Grandio composite in the use of different bonding agents based on qualitative analysis

	Clearfil SE Bond		G-Premio Bond with etching		Adper Single Bond 2		G-Bond		G-Premio Bond without etching		*p* Value
	No	%	No	%	No	%	No	%	No	%	
	9	30	22	73.3	14	50	7	23.3	10	33.3	0.004
1	16	53.3	6	20	13	43.3	13	43.3	13	43.4	
2	5	16.7	2	6.7	3	6.7	8	26.7	7	23.3	
3	0	0	0	0	0	0	2	6.7	0	0	

## Discussion

The present study assessed the microleakage of class-II composite restorations of primary molars using etch and rinse, self-etch, and multi-mode universal bonding agents. The dynamic environment of the oral cavity was simulated by exposing the samples to thermal changes via thermocycling, as 1500 thermal cycles between 5 and 55°C [ 10 ].

Both quantitative and qualitative methods were employed for the assessment and comparison of microleakage. In the quantitative method, image analysis software programs were used to measure the actual amount of microleakage. In the qualitative method, a scoring system from 0 to 3 was used. Some studies demonstrated a significant correlation between two methods and high levels of reliability for both [ 10 - 11 ]. 

Moreover, comparison of quantitative and qualitative results indicated a significant correlation between two methods. Therefore, both methods were applicable and could be referred to for evaluation.

Some controversies are present regarding the effect of self-etch bonding agents on the enamel [ 12 - 13 ]. Difference in the results of studies may be due to different confounding factors, such as the acidity of adhesives, different methodologies, and various bond strength tests. Some studies support the superior performance of more acidic systems [ 12 - 13 ], while Lopes *et al.* [ 14 ] showed that the bond strength of self-etch adhesives is dependent on the adhesive composition.

In the current study, no difference was observed between G-Premio Bond with or without etching, in comparison to Adper Single Bond 2 as the control group.

However, G-Premio bond with additional etching showed significantly lower microleakage than that observed in self-etch mode. In fact, the process of bonding to enamel etched with phosphoric acid is based on the micromechanical interlocking of adhesive into the porosities created by demineralization of enamel [ 3 ]. One of the shortcomings of self-etch adhesives, especially the universal adhesives when used in self-etch mode, is the reduction in the available enamel surface for a suitable bond compared to the use of phosphoric acid. This may vary depending on the adhesive pH [ 13 ].

In a systematic review, Rosa *et al.* [ 15 ] compared the bond strength of several universal adhesives and recommended selective etching of the enamel prior to the use of universal adhesives with mild etching property to enhance bond strength. However, with the use of universal adhesive with mild acidic property for bonding to dentin, no significant difference in bond strength between self-etch and etch and rinse modes was noted [ 15 ]. 

Yoshida *et al.* [ 16 - 17 ] were the first to suggest the concept of adhesive decalcification, who discussed the ability of stronger bond of 10-methacryloyloxydecyl dihydrogen phosphate (10-MDP) to synthetic hydroxyapatite compared to 4-methacryloxyethyl trimellitic (4-MET) and 2-methacryloxyethyl phenyl hydrogen phosphate (Phenyl-P). In fact, 10-MDP monomers enable the demineralization of dental hard tissue and ionic bond with calcium ions. G-Premio Bond universal adhesive contains 4-MET and 10-MDP functional monomers [ 18 ]. In some self-etch adhesives, 2-hydroxyethyl methacrylate (HEMA) is added to improve the wetting property and prevent the separation of hydrophobic resin components after evaporation of solvent. However, HEMA can keep water; decrease the degree of conversion, lead to hydrolytic degradation, and compromise long-term interface durability [ 19 ]. 

It has been shown that even insignificant amounts of HEMA can interfere with the chemical bonding of 10-MDP monomers to calcium ions in tooth structure [ 20 ]. As HEMA, according to the manufacturer, is not used in the formulation of G-Premio Universal adhesive, it can be stated that the elimination of this material from the formulation of this bonding improves the bond strength and decreases microleakage [ 18 ]. Furthermore, this universal adhesive has stronger acidity than other self-etch adhesives used in this study (moderate, pH of 1.5) [ 18 , 20 ]. The presence of 4-MET with 10-MDP in Self-Etch Bonding can significantly improve the bond strength compared to HEMA [ 21 ]. G-Bond (one-step self-etch) showed the highest microleakage compared to other bonding agents. This adhesive contains the 4-MET substance, which is an acidic monomer with a cyclic group and soluble in acetone. This combination leads to the formation of an ionic bond with the calcium present in hydroxyapatite and also is known as a demineralizing monomer, which results in the improvement of adhesion [ 22 ]. 

According to the manufacturer’s instruction, a monomeric phosphoric acid ester is used in combination with 4-methacryloxyethyl trimellitic acid (4-MET) to produce G-Bond that enhances etchant effectiveness and adhesion to enamel [ 18 ]. X-ray photoelectron spectroscopy (XPS) studies revealed that chemical bonding of 10-MDP with hydroxyapatite is stronger than that of 4-MET and is more stable in water [ 17 ]. 

Enamel's etching pattern of G-Bond is not a defined pattern, which is attributed to lower acidity of this adhesive. In addition, G-Bond results in higher enamel microleakage following thermal stresses and may be unable to penetrate through smear layers [ 23 ]. 

Despite the advantages of HEMA-free adhesives, the lack of HEMA may result in phase separation at the interface, previously observed with G-Bond, and compromise the bonding [ 19 ]. The presence of triethylene glycol dimethacrylate (TEGDMA) in G-Bond, which absorbs more amount of water after polymerization than bisphenol-glycidyl methacrylate (BIS-GMA), might explain its high microleakage score, phase separation and osmotic blistering [ 24 ].

Chandra *et al.* [ 25 ] showed that G-Bond failed to demonstrate a good performance in enamel margin compared to other self-etch bonding agents; although their study was conducted on permanent teeth, their results were in line with the results of the present study. 

Duddu *et al.* [ 26 ] assessed the efficacy of three one-step self-etch bonding agents in primary teeth and showed that G-Bond produced the highest microleakage. 

Totally, one-step self-etch adhesives need enough acidity to be able to demineralize enamel and penetrate dentin smear layers. Therefore, they have highly hydrophilic monomers, which make them liable to water degradation [ 27 ]. 

In the present study, in Clearfil SE Bond (two-step self-etch adhesive), microleakage was higher than that of G-Premio Bond (with etching) and Adper single bond 2. However, it was only significant compared to G-Premio Bond (with etching). The higher microleakage may be due to the presence of enamel margin and etching with phosphoric acid as a result of using the latter two adhesives. Despite the presence of 10-MDP, Clearfil bonding agent contains mild acidic primer (pH of 2.0); thus, it may be a reason for less effective bond with enamel. The combination of 10-MDP and 4-MET in the composition of G-Premio bond may be a reason for more durable bond.

Differences in hydroxyapatite structure in dentin and enamel can explain the interactive pattern of 10-MDP with these substrates. The smaller size and less amount of hydroxyapatite crystals, as well as cross-orientation of these crystals in dentin compared to enamel make dentin more receptive to form a chemical bond with 10-MDP [ 27 ]. However, the presence of 4-MET can improve this bond as explained previously [ 21 ]. 

Deliper *et al.* [ 1 ] showed that Clearfil SE Bond had higher microleakage in enamel margin compared to dentin margin and that the additional etching of enamel improved bonding quality. Therefore, it is recommended to etch the enamel surface prior to the application of this bonding agent. 

The occurrence of microleakage with the use of Adper Single Bond 2 (two-step etch-and-rinse) was significantly lower than that observed in other groups. However, the appearance of microleakage in this group was not significantly different from that of universal adhesive with etching. This adhesive is used after enamel surface conditioning with phosphoric acid, which has a highly acidic pH; this explains significantly lower microleakage in this group. It contains poly alkenoic acid copolymer (Vitrebond) in its composition and this functional methacrylate copolymer is a combination of poly-acrylic and polyitaconic acids that was first used in the composition of Vitrebond™ Glass Ionomer (3M ESPE). It has been suggested that Vitrebond copolymer is responsible for a chemical adhesion with hydroxyapatite [ 28 ]. 

There are few studies on the effect of self-etch adhesives on primary enamel or dentin; the aprismatic layer in primary enamel is thicker than that of permanent teeth and since this layer can interfere with acid etching pattern, bonding to primary enamel is weaker than that of permanent enamel [ 29 ]. This demonstrates the necessity of primary dentition conditioning by phosphoric acid to obtain a more durable bonding.

This study had an in vitro design and thus, had the limitations of such studies. In oral clinical settings, thermal, mechanical, and chemical stresses are present, which affect the occurrence of microleakage. In addition, fatigue test and bond strength test after long-term storage should be done to draw a firm conclusion on the selection of an ideal bonding agent. 

## Conclusion

None of the bonding systems could completely prevent microleakage. Use of universal adhesives in self-etch mode is suitable for composite restoration of primary teeth due to lower microleakage, fewer application steps, and easy use. Additional etching is recommended for the application of multi-purpose universal adhesives in enamel margins to improve the quality of bonding.

## Conflict of Interest

The authors declare that they have no conflicts of interests.
